# Trisomy 8 Associated Clonal Cytopenia Featured With Acquired Auto-Inflammation and Its Response to JAK Inhibitors

**DOI:** 10.3389/fmed.2022.895965

**Published:** 2022-04-25

**Authors:** Yakai Fu, Wanlong Wu, Zhiwei Chen, Liyang Gu, Xiaodong Wang, Shuang Ye

**Affiliations:** Department of Rheumatology, Renji Hospital, School of Medicine, Shanghai Jiao Tong University, Shanghai, China

**Keywords:** trisomy 8, auto-inflammation, myelodysplasia, clonal hematopoiesis, Janus kinase inhibitor

## Abstract

**Objects:**

It has been recognized the nexus between trisomy 8 and auto-inflammatory features in myelodysplasia syndrome (MDS). Recent research about VEXAS syndrome proved clonal hematopoiesis could interfere with innate immune system far before occurrence of hematological malignancies. We reported a case series of clonal cytopenia with auto-inflammatory features in trisomy 8 patients.

**Methods:**

A total of six patients with isolated trisomy 8 excluded from MDS was retrospectively collected from the Department of Rheumatology, Renji Hospital, Shanghai. The clinical presentations and treatment outcomes were presented.

**Results:**

We report patients with trisomy 8 shared the auto-inflammatory features of recurrent fever, arthralgia, gastrointestinal involvement, and elevated inflammatory markers, especially hyperferritinemia, in addition to hematological findings such as macrocytic anemia and cytopenia of other lineages but without myelodysplasia. The symptoms of this disorder responded to the treatment of glucocorticoids but difficult to taper. JAK inhibitors were introduced to four patients with enhanced response along with glucocorticoids sparing effect and good tolerance.

**Conclusion:**

Clonal cytopenia harboring trisomy 8 presenting with auto-inflammatory features was identified. JAK inhibitor may be a promising anti-inflammatory option.

## Introduction

Abnormal activation of innate immune system and its associated autoinflammation had been reported in recent studies in many myeloid neoplasms (1). Trisomy 8 is one of the most common cytogenetic abnormalities in myeloid neoplasms such as myelodysplasia syndrome (MDS) and acute myeloid leukemia (AML) (2, 3). The possible link between MDS with trisomy 8 (+8-MDS) and autoinflammatory condition, Behcet’s Disease (BD) in particular, has been well recognized in the past decades (4). However, Behcet’s like syndrome with +8-MDS differed from classic BD with more frequent gastrointestinal (GI) but less pseudofolliculitis or ocular involvement, along with prominent MDS hematologic features (5, 6). It is noteworthy that trisomy 8 *per se*, in the absence of diagnostic MDS morphological features, is not sufficient for a MDS diagnosis (7, 8). In previous studies, researchers focused on autoimmune or autoinflammatory features only in the confirmed +8-MDS, whereas the conditions in abnormal karyotypes without myelodysplasia had been largely overlooked.

Here, we described a case series of autoinflammatory syndrome associated with isolated +8 who were excluded from MDS. In addition, the treatment strategies of these six patients had also been discussed. Most patients responded to the glucocorticoids initially but flared in tapering. The possible efficacy of Janus kinase inhibitor for these patients was also exploited.

## Methods

We retrospectively collected patients received cytogenetics examination on bone marrow from 2014 to the present at the Rheumatology Department of Renji Hospital South Campus, Shanghai Jiao Tong University School of Medicine, Shanghai, China. Patients with isolated +8 karyotype and exclusion of other hematological disorders [according to 2016 WHO classification of myeloid neoplasms (9)], were included. Clinical and laboratory data were summarized and analyzed. The study followed local ethics committee regulations with informed consent obtained from all participants.

## Results

A total of seven patients with isolated +8 karyotype were identified. After morphologic evaluation of bone marrow, one patient was diagnosed as MDS-EB1 and excluded. The clinical characteristics of remaining six patients were presented in Table 1.

**TABLE 1:** The clinical and laboratory findings in patients.

	Case 1	Case 2	Case 3	Case 4	Case 5	Case 6
Age/sex	69/M	36/M	67/M	62/M	66/M	40/F
Symptoms	Fever, arthralgia	Fever, lower abdominal pain, arthralgia, diarrhea	Fever, arthralgia, abdominal distension	Fever, rash, arthralgia, abdominal pain	Fever, oral uclers abdominal pain, hematochezia	Fever, rash, erythema nodosum, abdominal pain, thrombosis
Gastrointestinal findings	None	Ileocecal ulcer and edema	Mesenteric panniculitis	Mesenteric panniculitis	Terminal ileum ulcer	Terminal ileal nercrosis
Past history	None	Immune thrombocytopenia purpura	None	None	None	Takayasu arteritis
WBC (*10^9^/L) (3.5–9.5)	5.17	3.22	3.77	6.11	4.89	3.04
Hb (g/L) (130–175)	92	66	117	79	63	100
MCV (g/L) (82–100)	112.8	120.1	116	127	108.2	111.2
MCH (pg) (27–34)	35.2	40.8	39.3	39.3	36.1	36.8
Plt (*10^9^/L) (125–350)	76	478	131	120	79	232
CRP (mg/L) (0–10)	139.6	101.74	18.34	165.66	150	194.9
ESR (mm/h) (0–15)	106	140	65	63	84	62
Ferrtin (ng/ml) (24–336)	1564	1605	2160	1134	861	1620.9
PCT (ng/ml) (0–0.1)	19.2	0.9	0.18	0.09	4.02	0.6
ANA	Negative	Negative	Negative	Negative	Negative	Negative
Additional gene mutation	*NLRP3* R675Q	\	*SF3B1* H662D	\	\	*U2AF1* S34Y
Previous treatment	GCs, MTX, colchicine	GCs	GCs, thalidomine	GCs, NSAIDs	GCs, 5-ASA	GCs, CTX, tocilizumab, tacrolimus
Last treatment	prednisone + tofacitinib	prednisone + tofacitinib	prednisone + tofacitinib	prednisone + thalidomine + CTX	prednisone + baritinib	prednisone + adalimumab
Follow-up time after JAKi (months)	22	6	8	\	4	\
Outcome	Improved	Improved	Improved with inflammation but progressed to MDS-RS	Loss of follow-up	Improved	Improved

*WBC, white blood cell; Hb, hemoglobin; MCV, mean corpuscular volume; MCH, mean corpuscular hemoglobin; Plt, platelet; CRP, C-reaction protein; ESR, erythrocyte sedimentation rate; PCT, procalcitonin; ANA, anti-nuclear antibody; GCs, glucocorticoids; MTX, methotrexate; NSAIDs, non-steroidal anti-inflammatory drugs; 5-ASA, 5-aminosalicylic acid; CTX, cyclophosphamide; MDS-RS, Myelodysplastic Syndrome with ring sideroblasts.*

### Case 1

**FIGURE 1:**
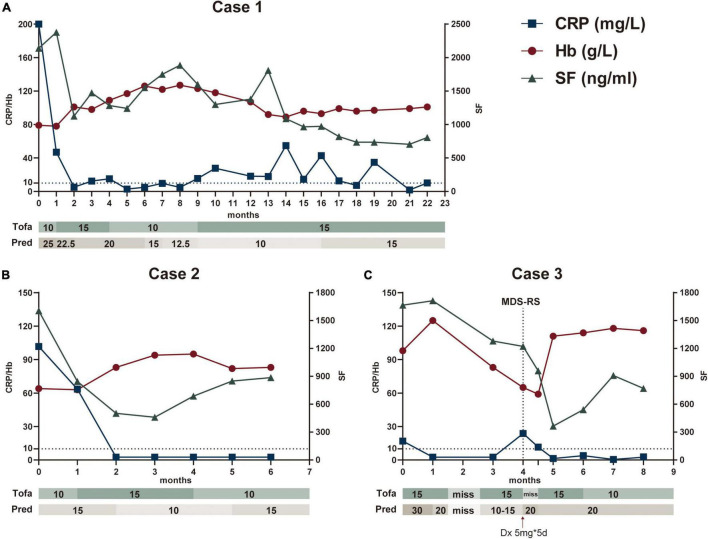
Main clinical and biologic findings and therapeutic interventions in three patients treated with tofacitinib. The Figures illustrated Case 1 **(A)**, Case 2 **(B)**, and Case 3 **(C)**, respectively. CRP and Hb referred to the left *Y* axis, SF referred to the right *Y* axis. CRP, C-reaction protein; Hb, hemoglobin; SF, serum ferritin; Tofa, tofacitinib (mg/day); Pred, prednisone (mg/day); Dx, dexamethasone; MDS-RS, Myelodysplastic Syndrome with ring sideroblasts.

A 69-year-old man reported recurrent fever and arthralgias without other accompanied symptom for 1 year. His C-reactive protein (CRP), procalcitonin (PCT), erythrocyte sedimentation rate (ESR), and serum ferritin (SF) levels were strikingly high, but evaluations for infectious (such as tuberculosis) or rheumatic conditions (such as giant cell arteritis) were unrevealing (Table 1). Peripheral blood analyses demonstrated macrocytic anemia (Hb 92 g/L and MCV 112.8 g/L) and mild thrombocytopenia (Plt 76*10^9^/L). Bone marrow (BM) examination showed no myelodysplasia but presence of +8. Additionally, a next-generation sequencing panel for autoinflammatory diseases was performed and found a heterozygous *NLRP3* R675Q missense mutation. Family history was unremarkable. This mutation of *NLRP3* was not reported to be pathogenic previously and identified as uncertain significance. Bioinformatics protein function prediction indicated this mutation was benign. A working diagnosis of autoinflammatory syndrome with trisomy 8 was established and the patient has a prompt response to dexamethasone 10 mg per day. Methotrexate 10 mg per week was subsequently added to facilitate glucocorticoids (GCs) tapering. However, fever and CRP resurged during reduction of GCs. Methotrexate was stopped and a therapeutic trial of tofacitinib 5 mg twice daily was initiated at the treatment of 25 mg prednisone per day, after discussion and agreement achieved with the patient. During the following 16-month follow up, tofacitinib was titrated to 15 mg/day and prednisone reduced to 15 mg per day as maintenance. Attempts to reduce either tofacitinib or prednisone to a lower dosage incurred flare of symptom or inflammatory markers. The patient remained stable and underwent regular follow up to date (Figure 1A).

### Case 2

A 36-year-old man, with a history of immune thrombocytopenia purpura 10 years ago and platelets recovered after splenectomy, who suffered from chronic anemia followed by intermittent fever and arthralgias for more than 1 year. The patient was suspected having autoimmune disease and received treatment of prednisone 15 mg per day with only suboptimal improvement. He developed lower abdominal pain and diarrhea 1 month prior to admission. No oral or ocular or genital lesions were presented. His CRP, PCT, ESR, and SF were elevated, along with macrocytic anemia (Hb 66 g/L and MCV 120.1 g/L) and mild leukopenia (WBC 3.22*10^9^/L). The autoantibody panel was negative. Despite of finding +8 in karyotype, hematological malignancy was not suggested by BM examination. Abdominal CT indicated ileocecal edema, and colonoscopy confirmed large solitary ulceration on ileocecal valve. A working diagnosis of intestinal Behcet-like syndrome with trisomy 8 was made. Tofacitinib 10 mg per day was added in the first month and increased to 15 mg/day in the following 3 months, while prednisone maintained in 15 mg/day in the beginning and tapered to 10 mg/day from the second month. Continued to be symptom free with stable inflammatory markers and improvement of anemia, the patient declined to have a colonoscopy reexamination and tofacitinib was reduced to 10 mg/day in the 4th month (Figure 1B).

### Case 3

A 67-year-old man with recurrent fever, arthralgias and abdominal pain, who was diagnosed as having ‘mesenteric panniculitis’ and treated with thalidomide for 1.5 years prior to the presentation. The symptoms recurred with elevated CRP, ESR, and SF. He received first BM examination due to mild leukopenia (WBC 3.77*10^9^/L) and macrocytic anemia (Hb 117 g/L and MCV 116 g/L). Karyotype showed +8 but no myelodysplasia was detected. He was prescribed prednisone 30 mg per day and thalidomide but with suboptimal response. Tofacitinib of 15 mg per day was added. The fever and arthralgia were improved. Inflammatory markers also decreased. Unfortunately, patient stopped all medications by himself due to a car accident which resulted in a ulna fracture A month later, his hemoglobin level reduced from 101 g/L to 59 g/L despite of resuming prednisone and tofacitinib treatments (Figure 1C). A second BM examination was performed 13 months after the first biopsy. Ring sideroblast comprised 15% of nucleated erythroid cells and *SF3B1* H662D mutation (Variant Allele Frequency, VAF 11.2%) was identified in BM cells. The diagnosis of MDS-RS was made, and hematology consultation decided no chemotherapy or demethylation therapy at this stage. Thus, he continued the anti-inflammatory treatment of prednisone and tofacitinib with a watchful follow up. His hemoglobin dramatically recovered to over 110 g/L and kept sustained remission in the following 3 months. Tofacitinib was declined to 10 mg/day with prednisone 20 mg/day in 6th month.

### Case 4

A 62-year-old man presented with recurrent episodes of fever accompanied by evanescent pink rashes, joint pain, intermittent abdominal pain, and painful erythema nodules on extremities (spontaneous subsided after fever episode) in the past 1 year. Systematic examination in another medical center was otherwise unrevealing except for increased ESR/CRP and “mesenteric panniculitis” indicated by abdominal CT. Treatment of prednisone 60 mg per day was initiated with symptoms relieved. However, fever recurred and was not responded to NSAIDs add-on when prednisone tapered to 30 mg per day. Repeat blood test in our hospital confirmed high ESR, CRP, SF, and negative ANAs. Macrocytic anemia (Hb 79 g/L and MCV 127 g/L) was detected but leukocytes and platelets were within normal ranges. No hematological neoplasia was found except isolated +8 in karyotype through BM examination. A skin nodule biopsy of lower extremity proved panniculitis. After treatment of methylprednisolone 40 mg per day and one dose of cyclophosphamide 0.6 g, symptoms were controlled. The patient was lost of follow-up after discharge.

### Case 5

**FIGURE 2:**
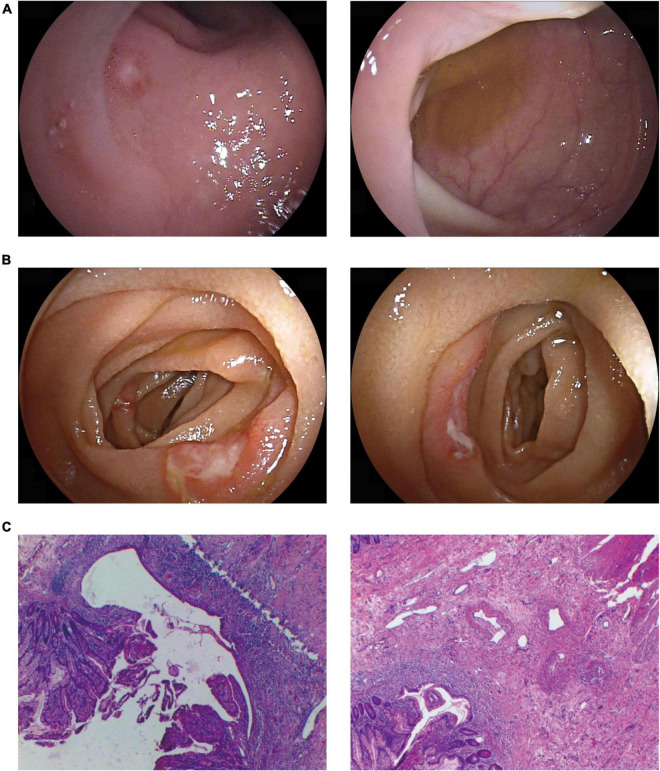
Representative gastrointestinal involvement in our cases. Typical endoscopic findings showed multiple ulcers of terminal ileum **(A)** and the lower jejunum **(B)**. Pathology suggested multiple shallow ulcers in the small intestinal mucosa **(C)**.

A 66-year-old man presented with intermittent fever, oral ulcers, and anemia for 6 months. He developed abdominal pain and hematochezia 1 month prior to admission. Laboratory investigations showed macrocytic anemia (Hb 63 g/L and MCV 108.2 g/L) with markedly increased CRP, ESR, PCT, and SF. The karyotype of trisomy 8 was detected but MDS was excluded by BM biopsy. No connective tissue disease, infection or malignancy was evident. Endoscopy was performed and showed multiple oval ulcers in lower jejunum and terminal ileum (Figures 2A,B). Biopsy revealed eosinophil infiltration in mucosa and submucosa and no epithelioid granuloma was found (Figure 2C). Methylprednisolone 40 mg per day was started followed by gradual reduction. Baricitinib 4 mg per day was added at the 4th month to facilitate GC tapering. The patient attained symptom-free with the hemoglobin recovered to 114 g/L (remained macrocytic), while inflammatory markers returned to normal at the 8th months. The maintenance dose of methylprednisolone was 4 mg/day.

### Case 6

A 40-year-old woman was reported with recurrent high fever with rash and elevated CRP for half a year. She was diagnosed as Takayasu arteritis in other medical center because of carotid and subclavian artery stenosis and increased metabolism in PET-CT. Therefore, she received CTX first but swift to tocilizumab in combination with glucocorticoids. 3 months later, the patient developed abdominal pain, which was considered as a bowel obstruction and underwent partial resection of the small bowel near the ileocecal region. Treatment of prednisone with tacrolimus was initiated but fever flared in steroid tapering. Subsequently, the patient was admitted to our hospital. Laboratory investigations showed obviously increased CRP and SF with slightly macrocytic anemia (Hb 100 g/L and MCV 111.2 g/L) and leukopenia (WBC 3.04*10^9^/L). BM biopsy indicated trisomy 8 and *U2AF1* S34Y mutation (VAF 36.4%) without myelodysplasias. Adalimumab was selected in combination with prednisone to better control gastrointestinal symptoms.

### Summary

We described a case series of six patients harboring sole trisomy 8 in BM cells without myelodysplasia to begin with. Most cases were male with an average age of 57 years at disease onset and shared the symptoms of recurrent non-infectious fever with arthralgia or rash. Five of them had gastrointestinal manifestations of which three had ileocecal ulcerations, while another two only revealed having “mesenteric panniculitis” by CT scan but otherwise unremarkable after extensive GI evaluations. All patients had significantly elevated inflammatory markers including CRP, ESR, and SF. The autoantibody penal was negative, such as rheumatoid factor, autoantibodies binding to citrullinated antigens, antinuclear antibody, extractable nuclear antigen, antineutrophil cytoplasmic antibodies, or antiphospholipid antibody. Notably, macrocytic anemia was detected with or without other cytopenia. The initial MDS finding in BM smear and biopsy was lacking, although one patient evolved into MDS over time. Most manifestations could be ameliorated by 0.5 mg/kg prednisone but relapse was common during GC tapering. Except for one patient who lost-to-followup and one treating with adalimumab, the rest 4 patients received a JAK inhibitor add-on (tofacitinib in 3 and baricitinib for 1), which in turn enhanced anti-inflammatory effect and facilitated GC tapering. A dose-dependent phenomenon was observed, i.e., 15 mg per day of tofacitinib might be more efficacious than 10 mg per day in certain patients. No alarming adverse event was observed during the follow-up period up to 22 months.

## Discussion

Autoinflammation refers to abnormal chronic systematic inflammation mediated by innated immune system in the absence of persistent infection stimuli (10, 11). Monogenic systemic autoinflammatory diseases (SAIDs) are considered prototype of autoinflammatory disorders in contrast to autoimmune diseases. More than 50 monogenic SAIDs have been identified in the past decades. The most common feature of these diseases is recurrent febrile episodes with dramatically increased acute phase reactants and various manifestations affecting mucocutaneous, gastrointestinal, and/or musculoskeletal system typically among pediatric patients (12, 13). Current opinions believe the pro-inflammatory cytokines produced and released by innate immune cells are responsible for autoinflammation (12); for example, NLRP3 inflammasome and IL-1β pathway are at the central place in the pathogenesis of classic SAIDs (14, 15).

As comparison, the discovery of VEXAS syndrome extends the understandings of SAIDs (16). It proved that somatic mutation restricted to hematopoietic stem and progenitor cells could induce late-onset autoinflammatory disease (16, 17). Thus, a new concept of hemato-inflammatory disease is proposed to define systematic inflammatory disease caused by somatic mutations in blood cells, which may progress toward to hematopoietic disorders (17, 18). Trisomy 8 is common in myelodysplasia neoplasms and has variable phenotype. Notably, sole +8 is neither sufficient nor necessary to induce leukemogenesis, or in other words, to be diagnostic for MDS (8, 9). Likewise, not all +8-MDS presented with autoinflammatory features; moreover, only a minority of +8-MDS showing BD-like disease, which indicates trisomy 8 is also not sufficient to cause autoinflammatory or BD-like phenotype. Interestingly, almost all BD-like disease occurred in +8-MDS differed from classical BD with less eye lesion but more intestinal (ileocecal predominant) inflammation (4, 17). The detailed contribution of +8 to both autoinflammation and myelodysplasia remained unclear and need further investigations.

**FIGURE 3:**
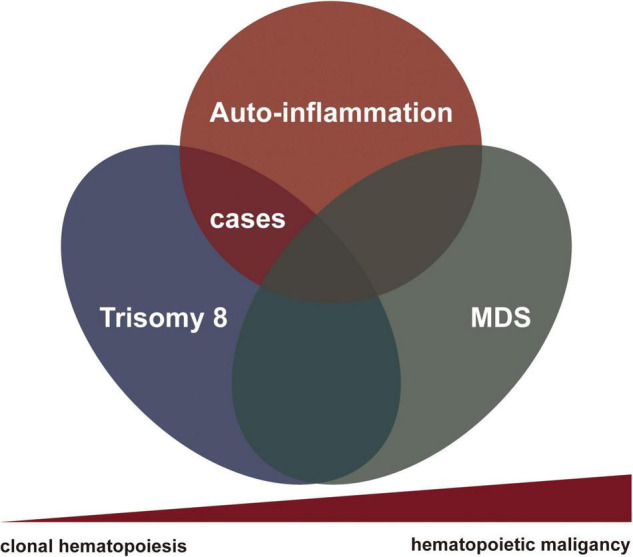
The nexus between rheumatological conditions and clonal hematopoiesis toward to myelodysplasia.

We herein summarized a small series of patients with hemato-inflammatory syndrome related to somatic trisomy 8 without myelodysplasia to begin with. All patients presented with macrocytic anemia and mild cytopenia involved other lineages; one patient eventually progressed to MDS-RS during the follow-up. It is likely that the syndrome represents an undifferentiated gray zone between autoinflammatory rheumatic condition and hematological disease (Figure 3). Clonal hematopoiesis could induce auto-inflammation far before it evolved into MDS or leukemia. Patients may suffer from severe clinical symptoms requiring active treatment before the settlement of diagnosis of hematological malignancies and the initiation of chemotherapy. It should be noted that only half of our patients received the gene mutation detection in BM cells or peripheral blood cells. A series of accompanying additional mutations with trisomy 8 were also identified. The contributions of these mutations to the clinical manifestations remain unclear. Futural detailed molecular examination was required to overcome this limitation.

The management of this rare autoinflammatory condition is tricky. Although still served as the most potent anti-inflammatory drug, glucocorticoid is always problematic especially with high-dose and long-term exposure. JAK inhibitors, on the other hand, might down-regulate multiple pro-inflammatory cytokines dependent on JAK/STAT signaling (19). The blockage of JAK has been proved to be promising in both SAIDs and myeloproliferative disorders with an established safety profiling (13, 19, 20). In our observations, JAK inhibitors displayed signals in terms of ameliorating systemic inflammations and facilitating glucocorticoids tapering without severe side effects in short terms. However, glucocorticoid seemed to be irreplaceable despite the combination of JAK inhibitors. No severe adverse event was reported with tofacitinib 15 mg/day, but the incident of adverse event of tofacitinib was related to go higher dosage. We attempted to keep tofacitinib in a regular dosage for safety precautions and pharmacoeconomic considerations, although 15 mg per day of tofacitinib might be more effective. Lager scale investigations and long-term follow up data are required to truly address the safety and efficacy of this rare syndrome in the future.

In conclusion, a new clonal cytopenia with autoinflammatory features characterized as recurrent fever, elevated inflammatory markers, and macrocytic anemia, with or without intestinal BD-like manifestations, is described harboring trisomy 8 in BM cells. JAK inhibitors might be a promising GC sparing drug to ameliorate the autoinflammatory symptoms.

## Data Availability Statement

The original contributions presented in the study are included in the article/supplementary material, further inquiries can be directed to the corresponding author.

## Ethics Statement

The studies involving human participants were reviewed and approved by the Ethics Committee, Renji Hospital, Shanghai Jiao Tong University School of Medicine. The patients/participants provided their written informed consent to participate in this study. Written informed consent was obtained from the individual(s) for the publication of any potentially identifiable images or data included in this article.

## Author Contributions

YF, WW, and SY contributed to the study design. YF, WW, ZC, and LG collected the clinical data. YF and SY wrote the manuscript. All authors have revised and agreed to the final version of the manuscript.

## Conflict of Interest

The authors declare that the research was conducted in the absence of any commercial or financial relationships that could be construed as a potential conflict of interest.

## Publisher’s Note

All claims expressed in this article are solely those of the authors and do not necessarily represent those of their affiliated organizations, or those of the publisher, the editors and the reviewers. Any product that may be evaluated in this article, or claim that may be made by its manufacturer, is not guaranteed or endorsed by the publisher.
